# Supplementary material for: Conductive carbon nanofiber interpenetrated graphene architecture for ultra-stable sodium ion battery
Source: Nat Commun. 2019 Sep 2;10:3917. doi: 10.1038/s41467-019-11925-z (PMC6718626; doi:10.1038/s41467-019-11925-z)
Supplement: Supplementary file 1 — Supplementary Information [file 41467_2019_11925_MOESM1_ESM.pdf]

Supporting information

Conductive Carbon Nanofiber Interpenetrated Graphene Architecture for Ultra-stable Sodium Ion Battery

Liu et al.

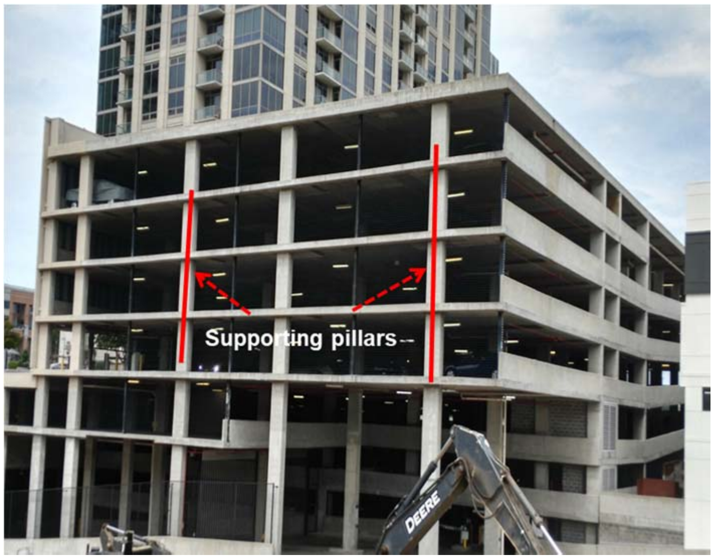

Supplementary Figure 1. Optical image of a construction building with greatly stable floors supported by various pillars.

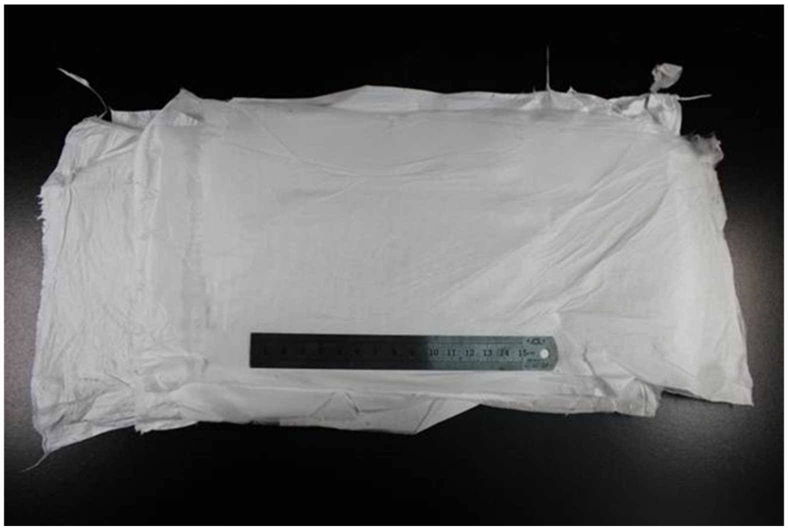

Supplementary Figure 2. Optical image of PAA fiber membranes.

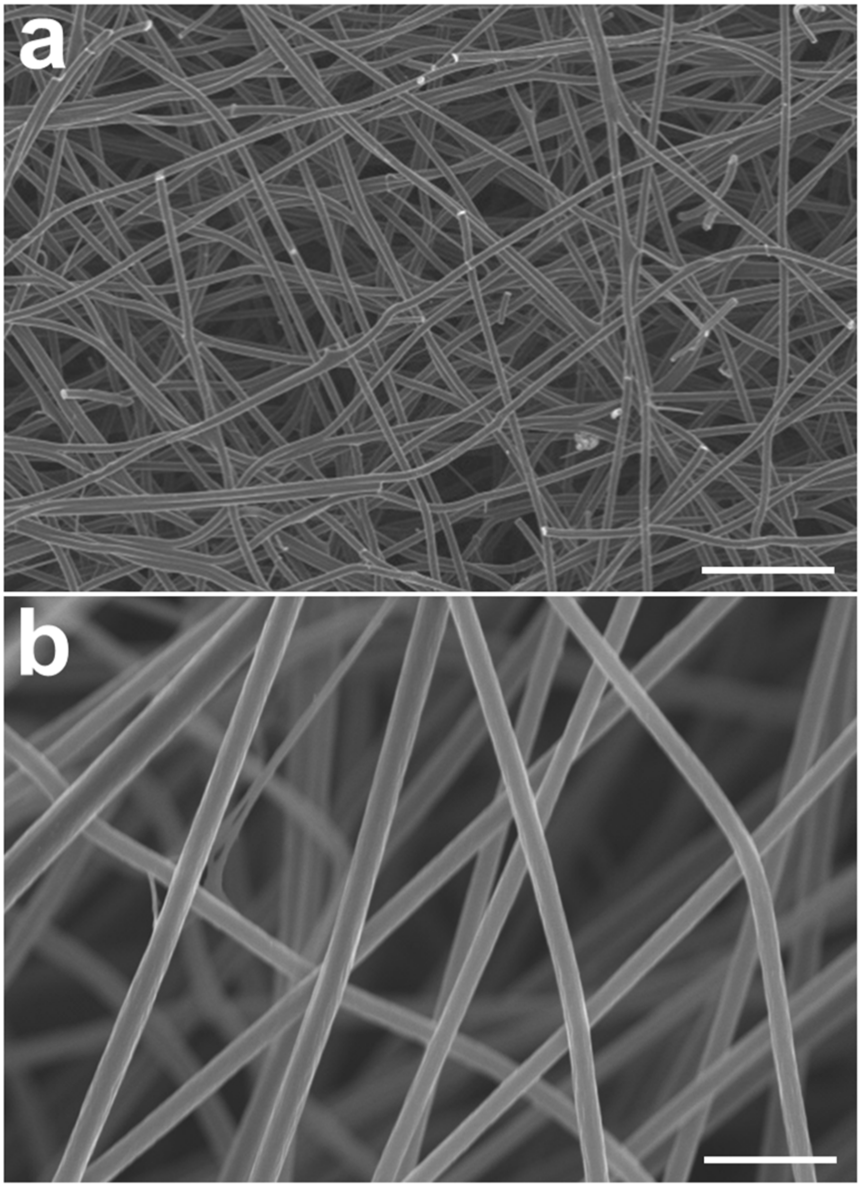

Supplementary Figure 3. **SEM images of carbon fiber membranes.** (a) low and (b) high magnifications of carbon fiber membrane. Scale bars: **a**. 20 μm, **b**. 5 μ 5.

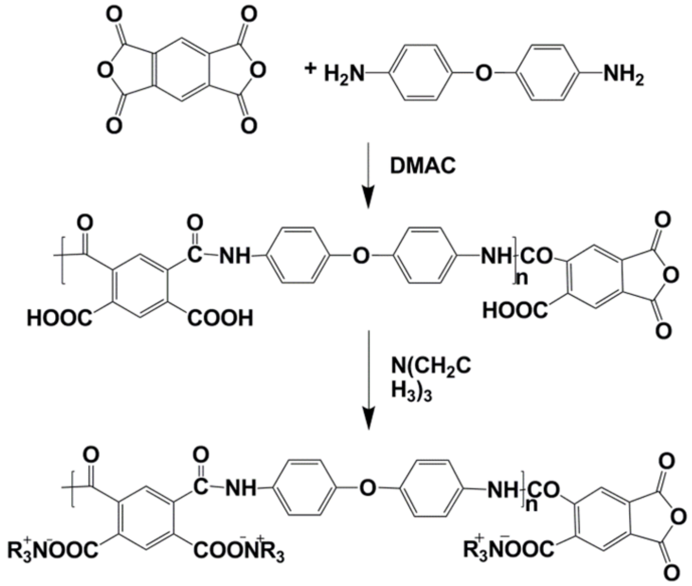

Supplementary Figure 4. Synthesis diagram of PAA materials based on ODA and PMDA.

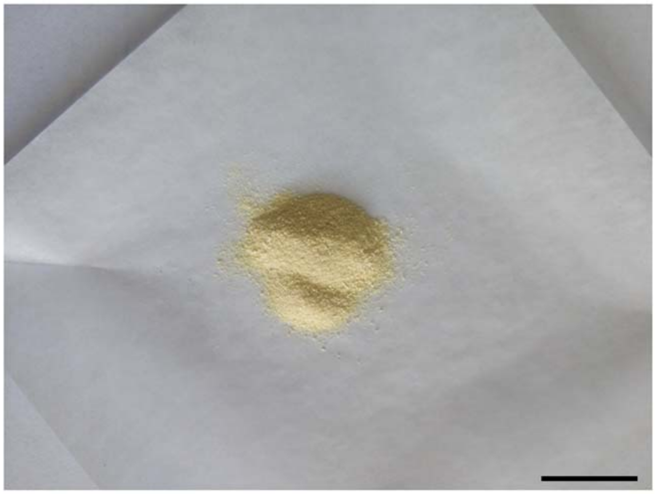

Supplementary Figure 5. Digital photo of PAA powder prepared based on the PAA-TEA solution with the assistance of freeze-drying treatment. Scale bar: 5 cm.

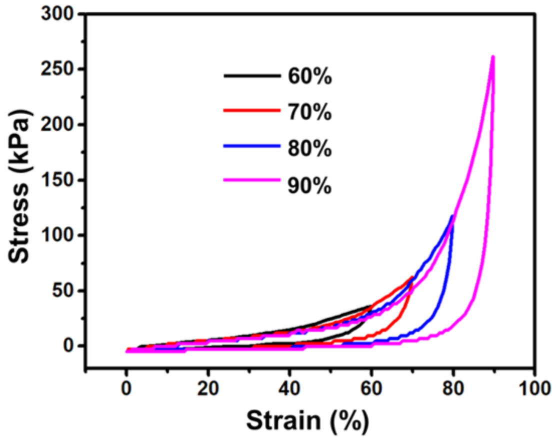

Supplementary Figure 6. Compressive stress-strain curves of CNFIG at different set strains of 60, 70, 80 and 90%.

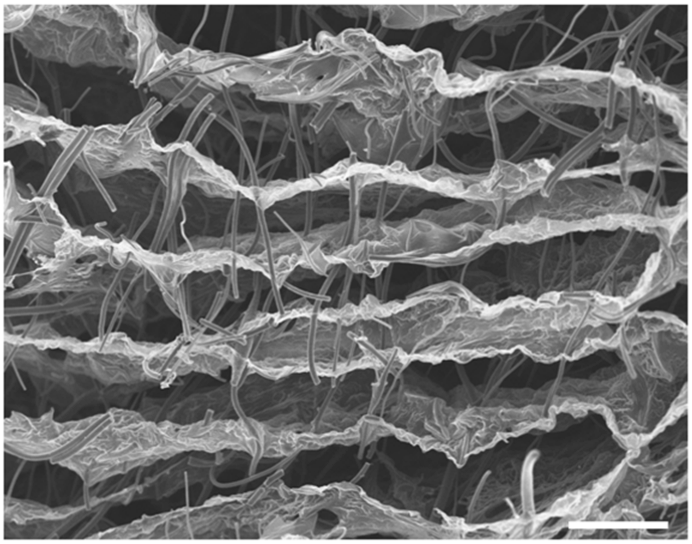

Supplementary Figure 7. SEM image of CNFIG aerogel at high magnification after being compressed for 100 times. Scale bar: 20 μm.

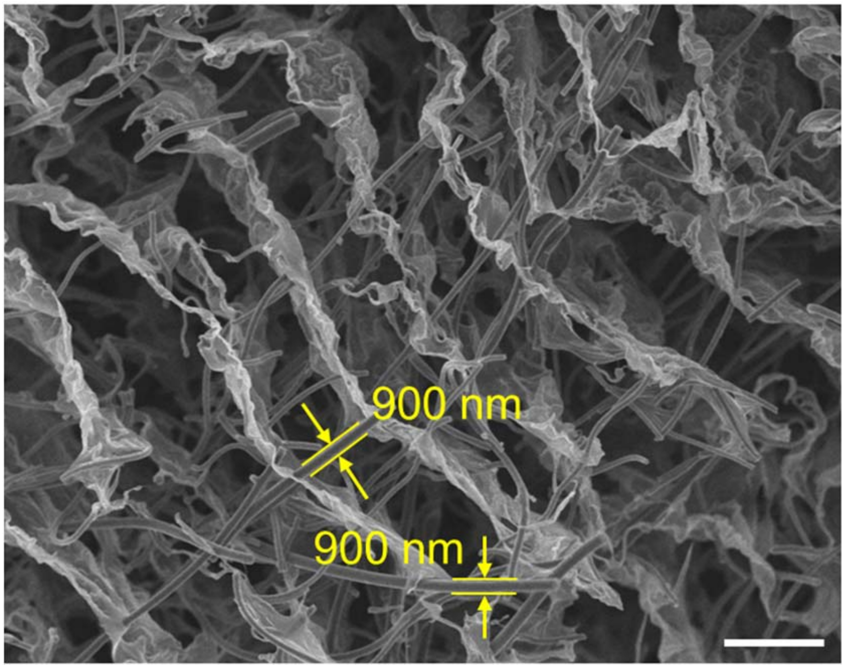

Supplementary Figure 8. SEM image of CNFIG aerogel consisting of CNFs with a larger diameter of 900 nm. Scale bar: 10 μm.

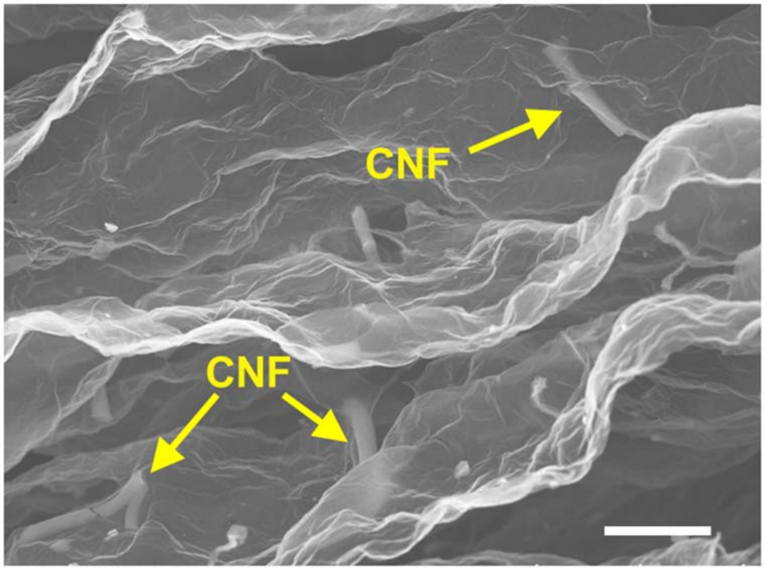

Supplementary Figure 9. SEM image of carbon aerogel based on GO@PAA@short CNF precursor at high magnification, in which the CNFs were only attached on the surface of carbon layers. Scale bar: 5 μm.

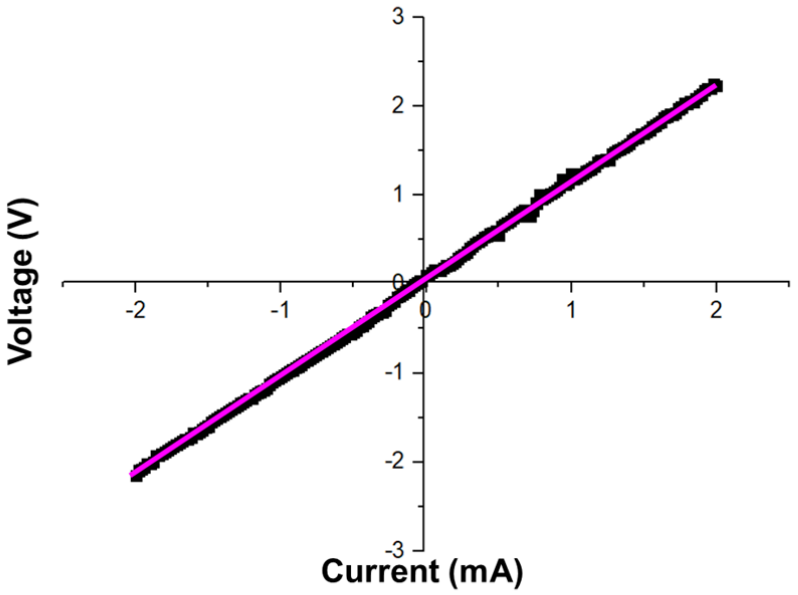

Supplementary Figure 10. Current-voltage curve of CNFIG matrix with a scan rate of 100 mV s^{-1}.

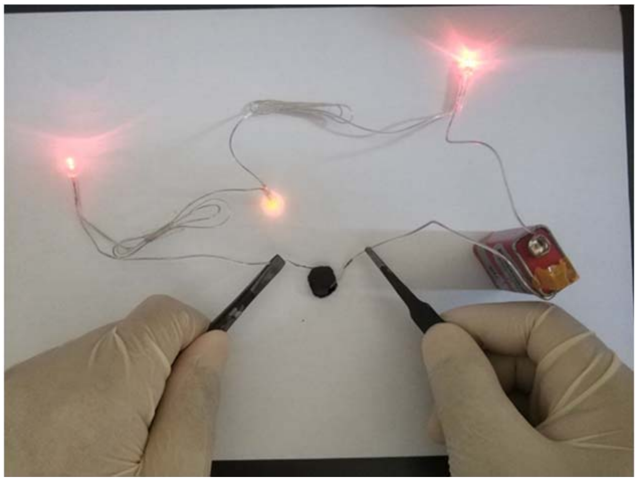

Supplementary Figure 11. A digital photo showing CNFIG matrix substituting the copper wire in a turn-on electrical circle.

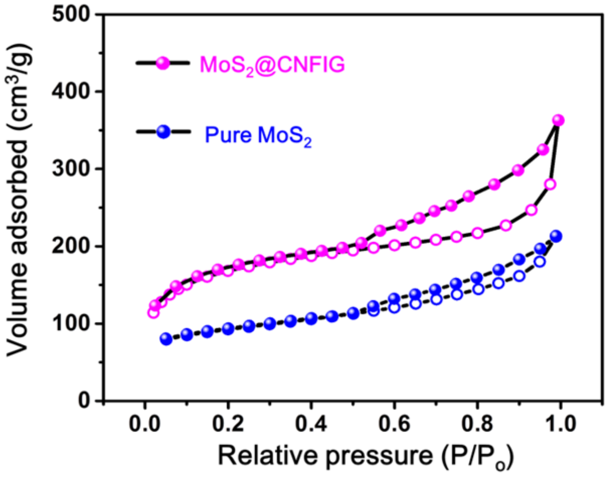

Supplementary Figure 12. Nitrogen adsorption-desorption isotherms of MoS_2@CNFIG hybrid and pure MoS_2 material.

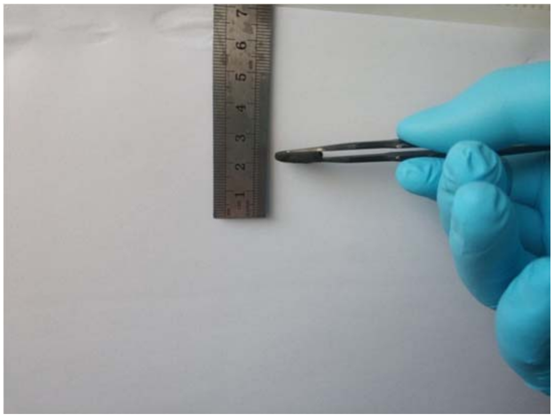

Supplementary Figure 13. Digital photo of MoS_2@CNFIG anode with a thickness of ~ 2.5 mm.

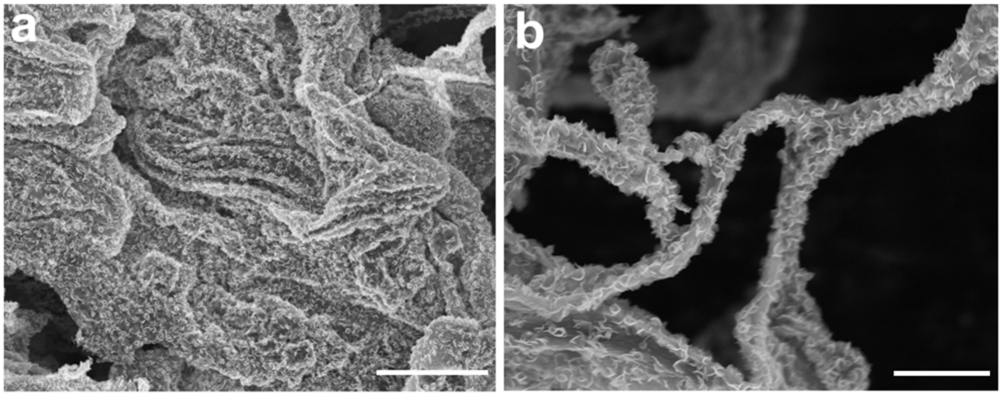

Supplementary Figure 14. SEM images of CNF/G/MoS_2 composite with randomly arranged structures at low and high magnifications. Scale bars: **a**. 5 µm, **b**. 2 µm.

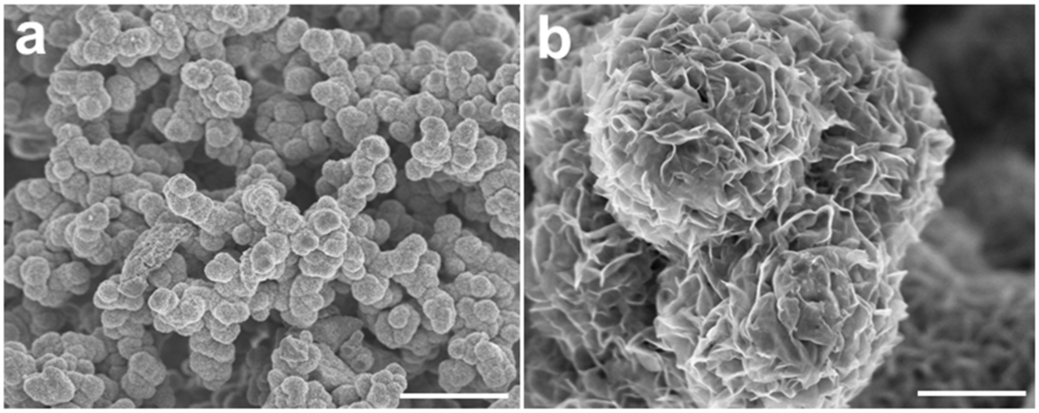

Supplementary Figure 15. SEM images of pure MoS_2 spheres at low and high magnifications. Scale bars: **a**. 20 μm, **b**. 5 μm.

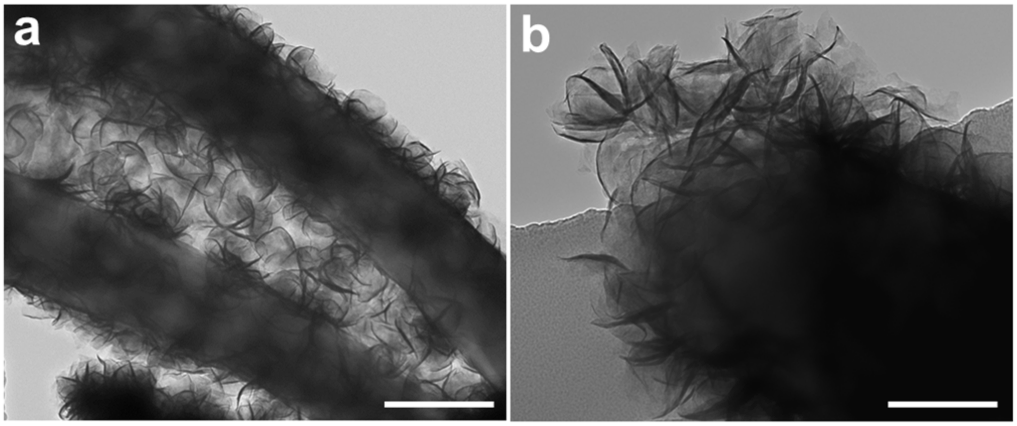

Supplementary Figure 16. TEM images of the MoS_2@CNFIG hybrid at (a) low and (b) high magnifications. Scale bars: **a**. 500 nm, **b**. 200 nm.

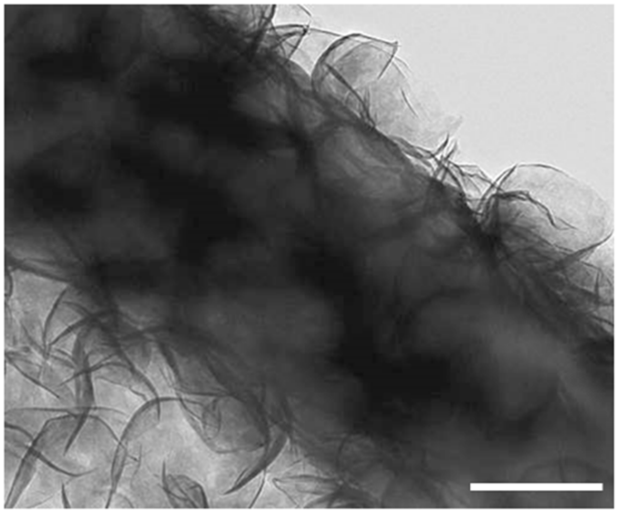

Supplementary Figure 17. TEM image of MoS_2@CNFIG hybrid at high magnification confirms the thickness of the anchored MoS_2 layer. Scale bar: 200 nm.

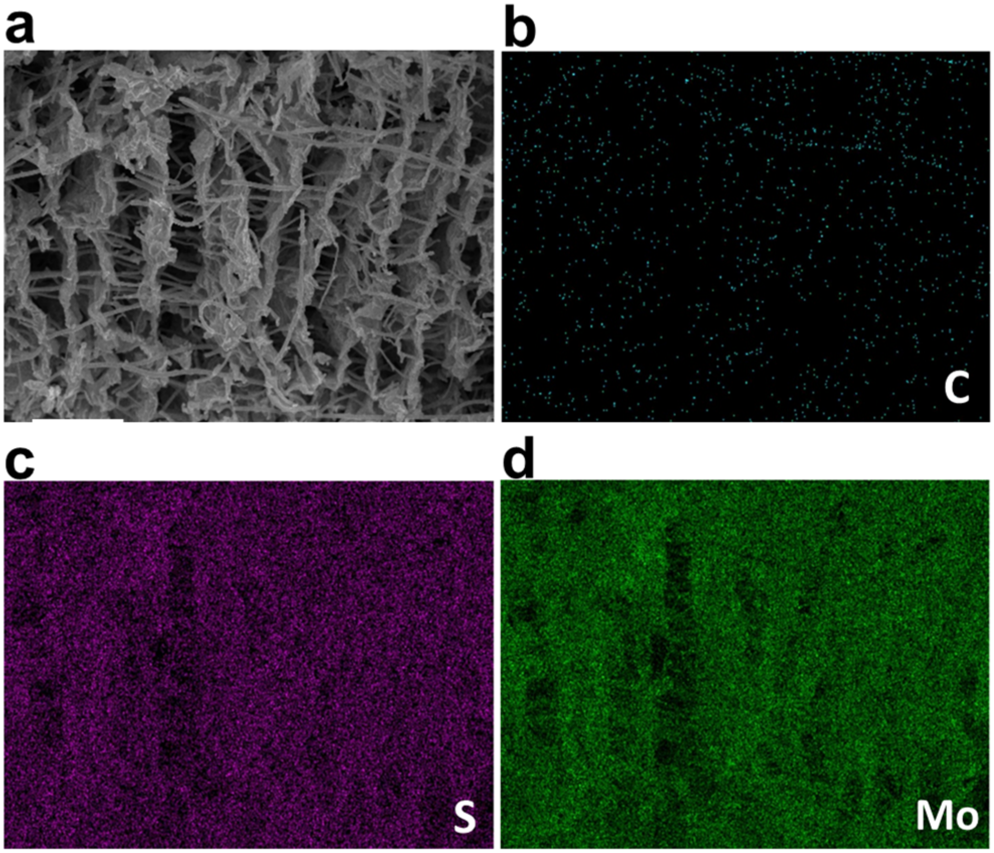

Supplementary Figure 18. **Elemental mappings of MoS_2@CNFIG.** (a) SEM image of MoS_2@CNFIG hybrid and (b - d) corresponding EDX elemental mappings of C, Mo and S, respectively; Scale bar: a. 40 μm.

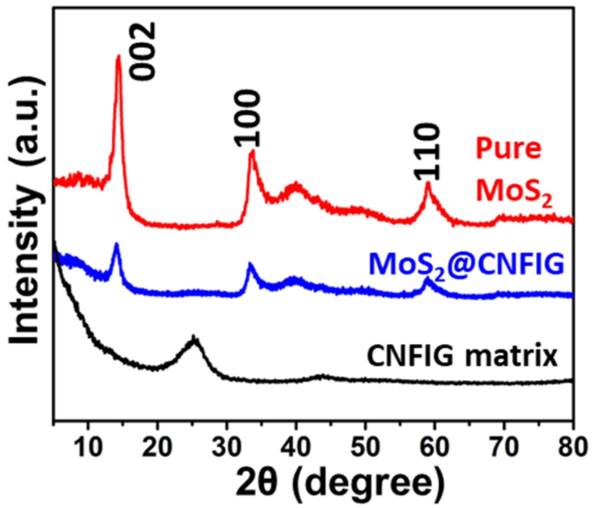

Supplementary Figure 19. XRD patterns of the pure MoS_2, CNFIG matrix, MoS_2@CNFIG hybrid.

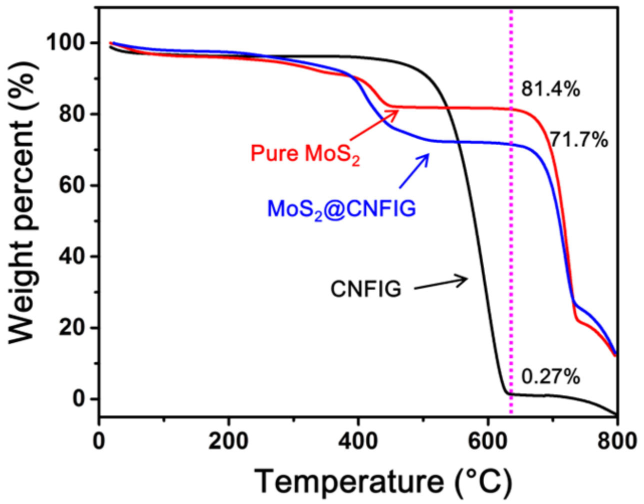

Supplementary Figure 20. TGA curves of pure MoS_2, CNFIG matrix and MoS_2@CNFIG composite in air at a heating rate of 10 ° C min^{-1} from room temperature to 800 ºC.

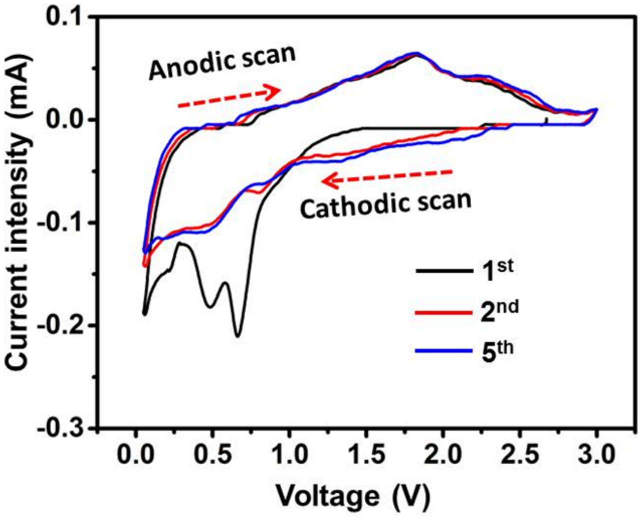

Supplementary Figure 21. CV curves of sodium ion cells with pure MoS_2 anode on the 1st, 2nd and 5th cycles.

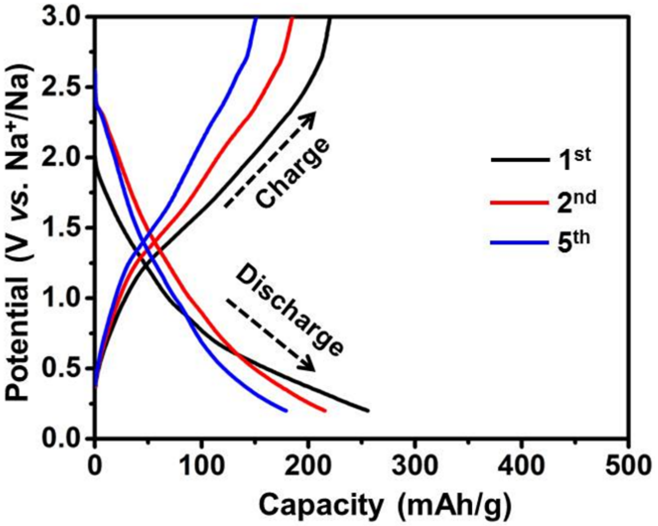

Supplementary Figure 22. Discharge/charge curves of sodium ion cells with pour MoS_2 anode on the 1st, 2nd and 5th cycles.

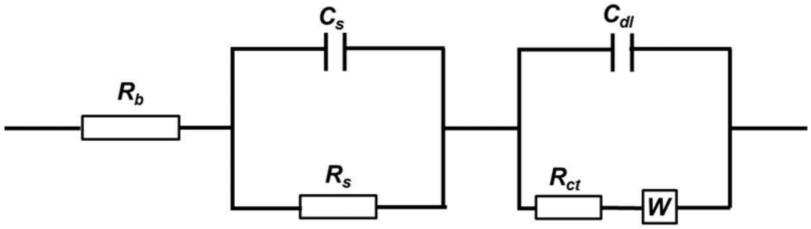

Supplementary Figure 23. Equivalent circuit used for fitting the EIS curves of pure MoS_2 and MoS_2@CNFIG hybrid.

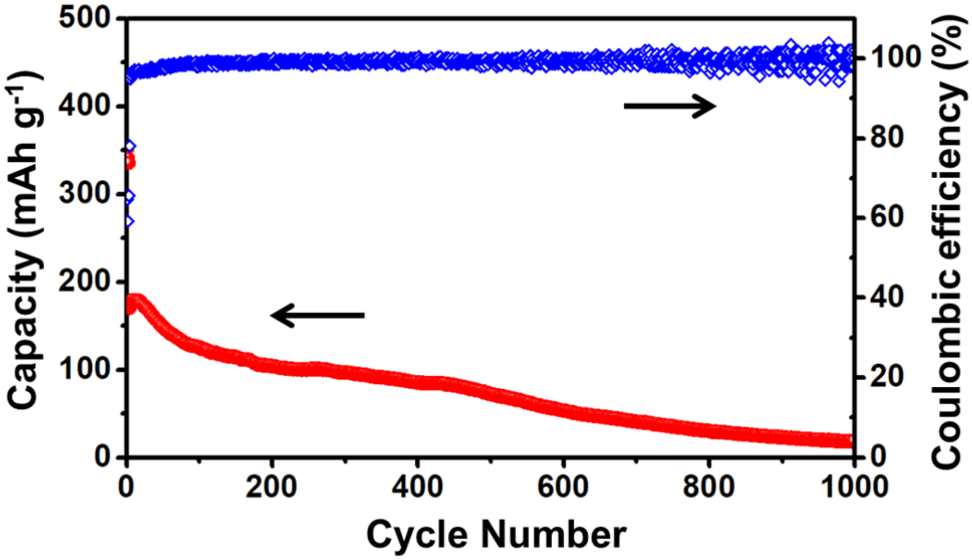

Supplementary Figure 24. Long-term cycling performance of pour MoS_2 anode at 1.0 A g^{-1} coupling with their Coulombic efficiencies.

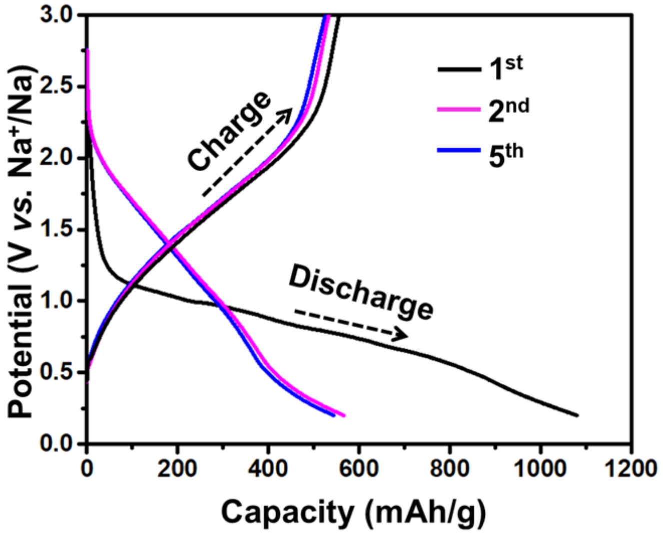

Supplementary Figure 25. Discharge/charge curves of CNF/G/MoS_2 composite on the 1st, 2nd and 5th cycles at 0.1 A g^{-1}.

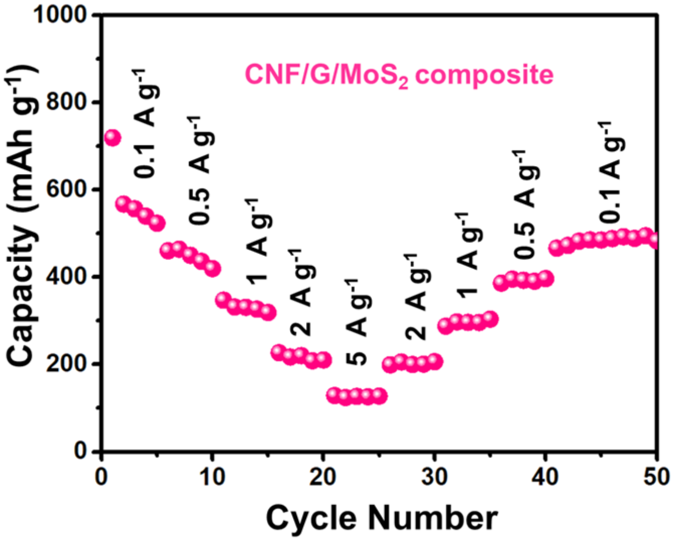

Supplementary Figure 26. Rate performances of CNF/G/MoS_2 composite at various current densities from 0.1 to 5.0 A g^{-1}.

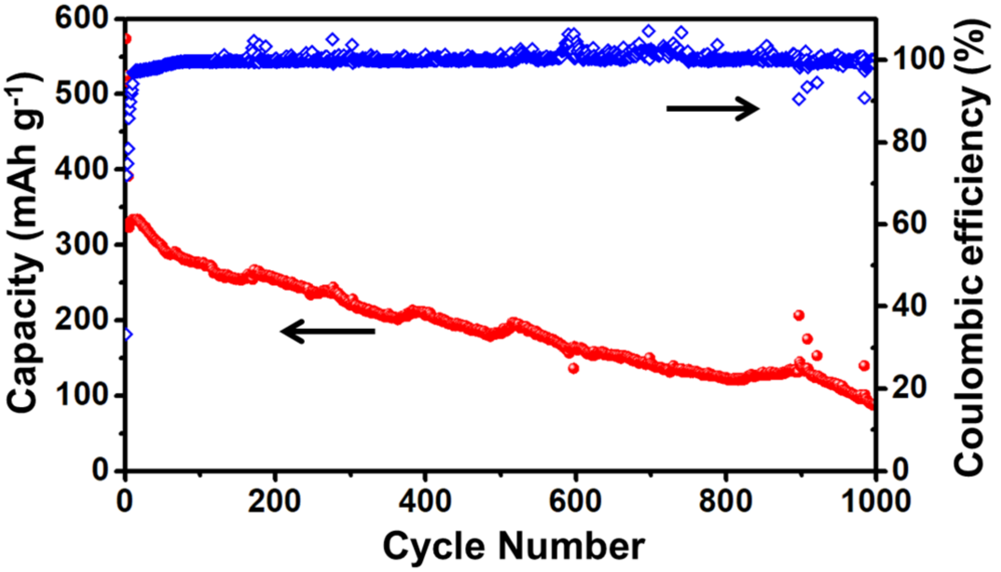

Supplementary Figure 27. Rate performances of CNF/G/MoS_2 composite at various current densities from 0.1 to 5.0 A g^{-1}.

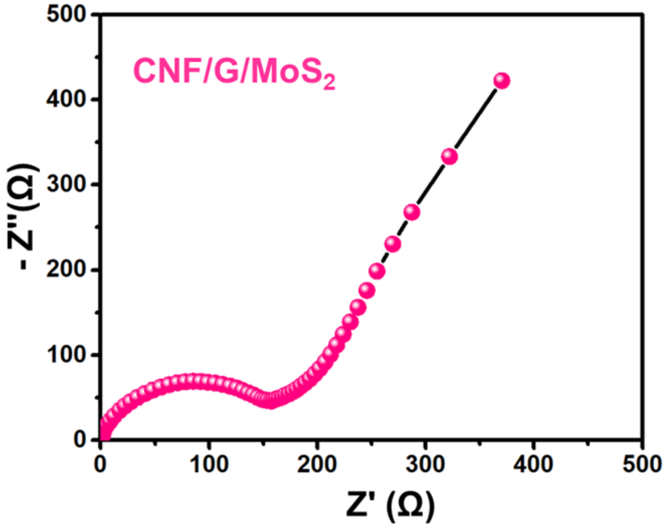

Supplementary Figure 28. EIS spectra of the fully charged CNF/G/MoS_2 composite anode in the frequency from 0.01 to 100 kHz.

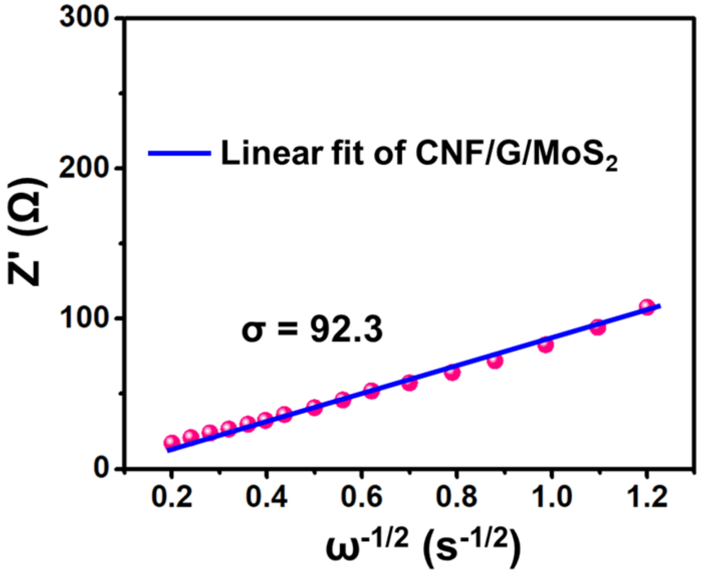

Supplementary Figure 29. The fitted Z' - $\omega^{-1/2}$ curves in the low-frequency region of EIS spectra for CNF/G/MoS_2 composite.

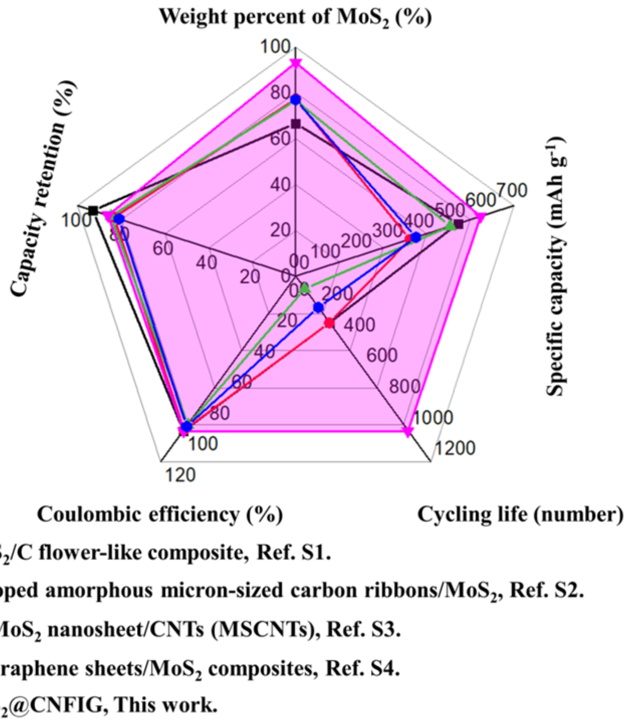

Supplementary Figure 30. Comparison of the electrochemical performances of MoS_2@CNFIG anode and the other kinds of MoS_2/carbon materials.

Supplementary References

1. Ahmed, B., Anjum, D. H., Hedhili, M. N. & Alshareef, H. N. Mechanistic Insight into the Stability of HfO_2-Coated MoS_2 Nanosheet Anodes for Sodium Ion Batteries. *Small* **11**, 4341-4350 (2015).
2. Pang, Y. *et al.* Few-layer MoS_2 anchored at nitrogen-doped carbon ribbons for sodium-ion battery anodes with high rate performance. *J. Mater. Chem. A* **5**, 17963-17972 (2017).
3. Zhang, S. *et al.* Growth of Ultrathin MoS_2 Nanosheets with Expanded Spacing of (002) Plane on Carbon Nanotubes for High-Performance Sodium-Ion Battery Anodes. *ACS Appl. Mater. Interfaces* **6**, 21880-21885 (2014).
4. Wang, Y.-X., Chou, S.-L., Wexler, D., Liu, H.-K. & Dou, S.-X. High-Performance Sodium-Ion Batteries and Sodium-Ion Pseudocapacitors Based on MoS_2/Graphene Composites. *Chem. Eur. J.* **20**, 9607-9612 (2014).
